# Supplementary material for: Overlapping cell population expression profiling and regulatory inference in C. elegans
Source: BMC Genomics. 2016 Feb 29;17:159. doi: 10.1186/s12864-016-2482-z (PMC4772325; doi:10.1186/s12864-016-2482-z)
Supplement: Additional file 13: — Web supplement. (DOC 21 kb) [file 12864_2016_2482_MOESM13_ESM.zip › sortWeb/clusters/index.html]

Cluster index 

# Cluster index

| cnd-1 8/19 | cnd-1 12/14 | cnd-1 1/4 | pha-4 5/9 | pha-4 9/1 | pha-4 12/9 | ceh-27 | ceh-36 | ceh-6 | F21D5.9 | mir-57 | mls-2 | pal-1 | pros-1 | ttx-3 | unc-130 | hlh-16 | irx-1 | ceh-6 (+) hlh-16 (+) | ceh-6 (+) hlh-16 (-) | ceh-6 (-) hlh-16 (+) | cnd-1 singlets | pha-4 singlets | t.000 | t.060 | t.120 | t.150 | t.180 | t.240 | t.330 | t.390 | t.420 | t.480 | t.540 | t.570 | t.600 | t.630 | t.660 | Cluster | Number of genes | Mean expr. (RPM) | Anatomy enriched | Other expression cluster | Motif | ChIP |
| --- | --- | --- | --- | --- | --- | --- | --- | --- | --- | --- | --- | --- | --- | --- | --- | --- | --- | --- | --- | --- | --- | --- | --- | --- | --- | --- | --- | --- | --- | --- | --- | --- | --- | --- | --- | --- | --- | --- | --- | --- | --- | --- | --- | --- |
|  |  |  |  |  |  |  |  |  |  |  |  |  |  |  |  |  |  |  |  |  |  |  |  |  |  |  |  |  |  |  |  |  |  |  |  |  |  | 1 | 40 | 131.1 |  | 2: 0hr muscle enriched... | 14: Nr2f6\_2 ... |  |
|  |  |  |  |  |  |  |  |  |  |  |  |  |  |  |  |  |  |  |  |  |  |  |  |  |  |  |  |  |  |  |  |  |  |  |  |  |  | 2 | 39 | 160 |  |  | 8: EMX1\_1 ... | 1: LIN-13\_Larvae-L4-stage... |
|  |  |  |  |  |  |  |  |  |  |  |  |  |  |  |  |  |  |  |  |  |  |  |  |  |  |  |  |  |  |  |  |  |  |  |  |  |  | 3 | 46 | 235.3 |  | 10: [cgc5428]:L1 muscle enriched... | 48: ARI3A\_do ... | 15: SAX-3\_Larvae-L3-stage... |
|  |  |  |  |  |  |  |  |  |  |  |  |  |  |  |  |  |  |  |  |  |  |  |  |  |  |  |  |  |  |  |  |  |  |  |  |  |  | 4 | 123 | 1526.5 |  | 45: [cgc5428]:L1 muscle enriched... | 128: pTH8649 ... | 131: LSY-2\_Larvae-L2-stage... |
|  |  |  |  |  |  |  |  |  |  |  |  |  |  |  |  |  |  |  |  |  |  |  |  |  |  |  |  |  |  |  |  |  |  |  |  |  |  | 5 | 137 | 215.6 |  | 23: TGF-beta adult downregulated... | 46: Zfp161\_2858 ... | 75: EFL-1\_Young-adult... |
|  |  |  |  |  |  |  |  |  |  |  |  |  |  |  |  |  |  |  |  |  |  |  |  |  |  |  |  |  |  |  |  |  |  |  |  |  |  | 6 | 63 | 188.2 |  | 3: L3 enriched... | 117: V$XFD1\_01 ... | 36: DPL-1\_Fed-L1-stage-larvae... |
|  |  |  |  |  |  |  |  |  |  |  |  |  |  |  |  |  |  |  |  |  |  |  |  |  |  |  |  |  |  |  |  |  |  |  |  |  |  | 7 | 138 | 0.3 |  |  | 55: pTH6447 ... |  |
|  |  |  |  |  |  |  |  |  |  |  |  |  |  |  |  |  |  |  |  |  |  |  |  |  |  |  |  |  |  |  |  |  |  |  |  |  |  | 8 | 27 | 58 |  |  | 17: pTH5074 ... |  |
|  |  |  |  |  |  |  |  |  |  |  |  |  |  |  |  |  |  |  |  |  |  |  |  |  |  |  |  |  |  |  |  |  |  |  |  |  |  | 9 | 41 | 21.7 |  |  | 2: gl\_SANGER\_5\_FBgn0004618 ... |  |
|  |  |  |  |  |  |  |  |  |  |  |  |  |  |  |  |  |  |  |  |  |  |  |  |  |  |  |  |  |  |  |  |  |  |  |  |  |  | 10 | 28 | 103.7 |  |  | 45: Hlf\_1 ... |  |
|  |  |  |  |  |  |  |  |  |  |  |  |  |  |  |  |  |  |  |  |  |  |  |  |  |  |  |  |  |  |  |  |  |  |  |  |  |  | 11 | 400 | 292.9 | 124: germ line... | 79: CYE-1 CDK-2AF upregulated... | 73: Zfp161\_2858 ... | 119: CEH-39\_Embryos... |
|  |  |  |  |  |  |  |  |  |  |  |  |  |  |  |  |  |  |  |  |  |  |  |  |  |  |  |  |  |  |  |  |  |  |  |  |  |  | 12 | 24 | 5.3 |  | 1: intestine CoreEnriched... | 3: pTH5919 ... |  |
|  |  |  |  |  |  |  |  |  |  |  |  |  |  |  |  |  |  |  |  |  |  |  |  |  |  |  |  |  |  |  |  |  |  |  |  |  |  | 13 | 15 | 3.1 |  |  |  |  |
|  |  |  |  |  |  |  |  |  |  |  |  |  |  |  |  |  |  |  |  |  |  |  |  |  |  |  |  |  |  |  |  |  |  |  |  |  |  | 14 | 18 | 8.1 |  |  | 3: pTH9300 ... |  |
|  |  |  |  |  |  |  |  |  |  |  |  |  |  |  |  |  |  |  |  |  |  |  |  |  |  |  |  |  |  |  |  |  |  |  |  |  |  | 15 | 23 | 0.3 |  |  | 1: Tin\_Cell\_FBgn0004110 ... |  |
|  |  |  |  |  |  |  |  |  |  |  |  |  |  |  |  |  |  |  |  |  |  |  |  |  |  |  |  |  |  |  |  |  |  |  |  |  |  | 16 | 89 | 0.5 |  |  |  |  |
|  |  |  |  |  |  |  |  |  |  |  |  |  |  |  |  |  |  |  |  |  |  |  |  |  |  |  |  |  |  |  |  |  |  |  |  |  |  | 17 | 125 | 0.6 |  |  | 1: pTH5437 ... |  |
|  |  |  |  |  |  |  |  |  |  |  |  |  |  |  |  |  |  |  |  |  |  |  |  |  |  |  |  |  |  |  |  |  |  |  |  |  |  | 18 | 59 | 1.5 |  | 5: PA14 vs OP50 upregulated 4hr... | 7: K562\_GATA2\_UChicago ... | 1: FOS-1\_Larvae-L2-stage... |
|  |  |  |  |  |  |  |  |  |  |  |  |  |  |  |  |  |  |  |  |  |  |  |  |  |  |  |  |  |  |  |  |  |  |  |  |  |  | 19 | 40 | 1.9 |  |  | 5: FLI1\_2 ... |  |
|  |  |  |  |  |  |  |  |  |  |  |  |  |  |  |  |  |  |  |  |  |  |  |  |  |  |  |  |  |  |  |  |  |  |  |  |  |  | 20 | 72 | 22.1 |  |  | 35: MA0047.2 ... |  |
|  |  |  |  |  |  |  |  |  |  |  |  |  |  |  |  |  |  |  |  |  |  |  |  |  |  |  |  |  |  |  |  |  |  |  |  |  |  | 21 | 55 | 1.6 |  |  | 2: Irx5\_2385 ... | 1: HLH-30\_Larvae-L4-stage... |
|  |  |  |  |  |  |  |  |  |  |  |  |  |  |  |  |  |  |  |  |  |  |  |  |  |  |  |  |  |  |  |  |  |  |  |  |  |  | 22 | 76 | 0.2 |  |  |  |  |
|  |  |  |  |  |  |  |  |  |  |  |  |  |  |  |  |  |  |  |  |  |  |  |  |  |  |  |  |  |  |  |  |  |  |  |  |  |  | 23 | 60 | 0.4 |  |  |  |  |
|  |  |  |  |  |  |  |  |  |  |  |  |  |  |  |  |  |  |  |  |  |  |  |  |  |  |  |  |  |  |  |  |  |  |  |  |  |  | 24 | 11 | 0.4 |  |  |  |  |
|  |  |  |  |  |  |  |  |  |  |  |  |  |  |  |  |  |  |  |  |  |  |  |  |  |  |  |  |  |  |  |  |  |  |  |  |  |  | 25 | 34 | 0.8 |  |  | 12: NKX22\_si ... |  |
|  |  |  |  |  |  |  |  |  |  |  |  |  |  |  |  |  |  |  |  |  |  |  |  |  |  |  |  |  |  |  |  |  |  |  |  |  |  | 26 | 151 | 27.3 |  |  | 108: pTH5661 ... | 1: EOR-1\_Larvae-L3-stage... |
|  |  |  |  |  |  |  |  |  |  |  |  |  |  |  |  |  |  |  |  |  |  |  |  |  |  |  |  |  |  |  |  |  |  |  |  |  |  | 27 | 32 | 5.5 |  |  |  |  |
|  |  |  |  |  |  |  |  |  |  |  |  |  |  |  |  |  |  |  |  |  |  |  |  |  |  |  |  |  |  |  |  |  |  |  |  |  |  | 28 | 31 | 0.9 |  |  | 4: NR4A2\_si ... | 1: ELT-3\_Embryos... |
|  |  |  |  |  |  |  |  |  |  |  |  |  |  |  |  |  |  |  |  |  |  |  |  |  |  |  |  |  |  |  |  |  |  |  |  |  |  | 29 | 20 | 0.3 |  |  | 7: V$HFH8\_01 ... |  |
|  |  |  |  |  |  |  |  |  |  |  |  |  |  |  |  |  |  |  |  |  |  |  |  |  |  |  |  |  |  |  |  |  |  |  |  |  |  | 30 | 79 | 220.1 |  | 5: coelomocytes embryo selectively enriched... | 145: Foxj3\_0982 ... |  |
|  |  |  |  |  |  |  |  |  |  |  |  |  |  |  |  |  |  |  |  |  |  |  |  |  |  |  |  |  |  |  |  |  |  |  |  |  |  | 31 | 428 | 53.8 |  |  | 295: TBP\_f1 ... |  |
|  |  |  |  |  |  |  |  |  |  |  |  |  |  |  |  |  |  |  |  |  |  |  |  |  |  |  |  |  |  |  |  |  |  |  |  |  |  | 32 | 103 | 109.5 |  |  | 11: HepG2\_SRF\_HudsonAlpha ... |  |
|  |  |  |  |  |  |  |  |  |  |  |  |  |  |  |  |  |  |  |  |  |  |  |  |  |  |  |  |  |  |  |  |  |  |  |  |  |  | 33 | 49 | 2.2 |  |  | 7: HLH4C\_da\_SANGER\_5\_3\_FBgn0011277 ... |  |
|  |  |  |  |  |  |  |  |  |  |  |  |  |  |  |  |  |  |  |  |  |  |  |  |  |  |  |  |  |  |  |  |  |  |  |  |  |  | 34 | 18 | 37.3 |  |  |  |  |
|  |  |  |  |  |  |  |  |  |  |  |  |  |  |  |  |  |  |  |  |  |  |  |  |  |  |  |  |  |  |  |  |  |  |  |  |  |  | 35 | 49 | 120 |  | 1: BAG-neuron embryo selectively enriched... | 135: pTH9385 ... |  |
|  |  |  |  |  |  |  |  |  |  |  |  |  |  |  |  |  |  |  |  |  |  |  |  |  |  |  |  |  |  |  |  |  |  |  |  |  |  | 36 | 35 | 22.3 |  |  | 8: pTH9215 ... |  |
|  |  |  |  |  |  |  |  |  |  |  |  |  |  |  |  |  |  |  |  |  |  |  |  |  |  |  |  |  |  |  |  |  |  |  |  |  |  | 37 | 46 | 3.6 |  | 1: fer-1 upregulated... | 3: SOX2\_1 ... |  |
|  |  |  |  |  |  |  |  |  |  |  |  |  |  |  |  |  |  |  |  |  |  |  |  |  |  |  |  |  |  |  |  |  |  |  |  |  |  | 38 | 49 | 12.9 |  |  | 2: V$XFD3\_01 ... |  |
|  |  |  |  |  |  |  |  |  |  |  |  |  |  |  |  |  |  |  |  |  |  |  |  |  |  |  |  |  |  |  |  |  |  |  |  |  |  | 39 | 184 | 162.9 |  | 14: total muscle depleted... | 1419: MA0446.1 ... | 26: PHA-4\_Larvae-L2-stage... |
|  |  |  |  |  |  |  |  |  |  |  |  |  |  |  |  |  |  |  |  |  |  |  |  |  |  |  |  |  |  |  |  |  |  |  |  |  |  | 40 | 29 | 2 |  |  | 3: KLF6\_si ... |  |
|  |  |  |  |  |  |  |  |  |  |  |  |  |  |  |  |  |  |  |  |  |  |  |  |  |  |  |  |  |  |  |  |  |  |  |  |  |  | 41 | 37 | 14.4 |  |  | 11: MA0296.1 ... |  |
|  |  |  |  |  |  |  |  |  |  |  |  |  |  |  |  |  |  |  |  |  |  |  |  |  |  |  |  |  |  |  |  |  |  |  |  |  |  | 42 | 22 | 201.4 |  |  | 14: pTH9969 ... |  |
|  |  |  |  |  |  |  |  |  |  |  |  |  |  |  |  |  |  |  |  |  |  |  |  |  |  |  |  |  |  |  |  |  |  |  |  |  |  | 43 | 82 | 12.2 |  | 8: HQ 3d 2.0mM Up... | 29: pTH9877 ... | 3: NHR-28\_Larvae-L4-stage... |
|  |  |  |  |  |  |  |  |  |  |  |  |  |  |  |  |  |  |  |  |  |  |  |  |  |  |  |  |  |  |  |  |  |  |  |  |  |  | 44 | 135 | 111.6 |  |  | 262: pTH5267 ... |  |
|  |  |  |  |  |  |  |  |  |  |  |  |  |  |  |  |  |  |  |  |  |  |  |  |  |  |  |  |  |  |  |  |  |  |  |  |  |  | 45 | 281 | 88.9 |  | 5: mir-243 up regulated... | 526: ECC-1\_FOXA1\_HudsonAlpha ... | 2: PHA-4\_Larvae-L2-stage... |
|  |  |  |  |  |  |  |  |  |  |  |  |  |  |  |  |  |  |  |  |  |  |  |  |  |  |  |  |  |  |  |  |  |  |  |  |  |  | 46 | 275 | 189 | 4: pharynx... | 15: mir-243 up regulated... | 1807: HepG2\_FOXA2\_HudsonAlpha ... | 11: PHA-4\_Larvae-L2-stage... |
|  |  |  |  |  |  |  |  |  |  |  |  |  |  |  |  |  |  |  |  |  |  |  |  |  |  |  |  |  |  |  |  |  |  |  |  |  |  | 47 | 11 | 0.8 |  |  | 1: pTH5737 ... |  |
|  |  |  |  |  |  |  |  |  |  |  |  |  |  |  |  |  |  |  |  |  |  |  |  |  |  |  |  |  |  |  |  |  |  |  |  |  |  | 48 | 41 | 5.2 |  |  | 10: ELF1\_f1 ... | 1: EFL-1\_Fed-L1-stage-larvae... |
|  |  |  |  |  |  |  |  |  |  |  |  |  |  |  |  |  |  |  |  |  |  |  |  |  |  |  |  |  |  |  |  |  |  |  |  |  |  | 49 | 24 | 4.8 |  |  |  |  |
|  |  |  |  |  |  |  |  |  |  |  |  |  |  |  |  |  |  |  |  |  |  |  |  |  |  |  |  |  |  |  |  |  |  |  |  |  |  | 50 | 97 | 0.1 |  |  | 154: pTH9135 ... |  |
|  |  |  |  |  |  |  |  |  |  |  |  |  |  |  |  |  |  |  |  |  |  |  |  |  |  |  |  |  |  |  |  |  |  |  |  |  |  | 51 | 20 | 185.7 |  |  | 8: pTH5260 ... | 16: LIN-15B\_Larvae-L4-stage... |
|  |  |  |  |  |  |  |  |  |  |  |  |  |  |  |  |  |  |  |  |  |  |  |  |  |  |  |  |  |  |  |  |  |  |  |  |  |  | 52 | 177 | 408.5 | 1: Pharyngeal gland cell... | 3: lin-15B(n744) upregulated... | 67: Mv69 ... |  |
|  |  |  |  |  |  |  |  |  |  |  |  |  |  |  |  |  |  |  |  |  |  |  |  |  |  |  |  |  |  |  |  |  |  |  |  |  |  | 53 | 29 | 18.7 |  |  | 8: EGR3\_f1 ... |  |
|  |  |  |  |  |  |  |  |  |  |  |  |  |  |  |  |  |  |  |  |  |  |  |  |  |  |  |  |  |  |  |  |  |  |  |  |  |  | 54 | 25 | 6.4 |  |  | 2: RFX4\_2 ... |  |
|  |  |  |  |  |  |  |  |  |  |  |  |  |  |  |  |  |  |  |  |  |  |  |  |  |  |  |  |  |  |  |  |  |  |  |  |  |  | 55 | 30 | 83.3 |  |  | 9: OLIG2\_2 ... |  |
|  |  |  |  |  |  |  |  |  |  |  |  |  |  |  |  |  |  |  |  |  |  |  |  |  |  |  |  |  |  |  |  |  |  |  |  |  |  | 56 | 109 | 1105.3 |  | 24: WBPaper00025032:cluster 10... | 199: pTH10810 ... | 136: LIN-15B\_Larvae-L4-stage... |
|  |  |  |  |  |  |  |  |  |  |  |  |  |  |  |  |  |  |  |  |  |  |  |  |  |  |  |  |  |  |  |  |  |  |  |  |  |  | 57 | 29 | 3.7 |  |  |  |  |
|  |  |  |  |  |  |  |  |  |  |  |  |  |  |  |  |  |  |  |  |  |  |  |  |  |  |  |  |  |  |  |  |  |  |  |  |  |  | 58 | 22 | 1.3 |  |  | 6: V$RFX1\_01 ... |  |
|  |  |  |  |  |  |  |  |  |  |  |  |  |  |  |  |  |  |  |  |  |  |  |  |  |  |  |  |  |  |  |  |  |  |  |  |  |  | 59 | 16 | 6.5 |  |  | 8: Pax6\_3838 ... |  |
|  |  |  |  |  |  |  |  |  |  |  |  |  |  |  |  |  |  |  |  |  |  |  |  |  |  |  |  |  |  |  |  |  |  |  |  |  |  | 60 | 26 | 0.5 |  |  | 7: FLI1\_2 ... |  |
|  |  |  |  |  |  |  |  |  |  |  |  |  |  |  |  |  |  |  |  |  |  |  |  |  |  |  |  |  |  |  |  |  |  |  |  |  |  | 61 | 168 | 0.2 |  |  | 8: V$EN1\_01 ... |  |
|  |  |  |  |  |  |  |  |  |  |  |  |  |  |  |  |  |  |  |  |  |  |  |  |  |  |  |  |  |  |  |  |  |  |  |  |  |  | 62 | 283 | 0.8 |  |  | 30: pTH6140 ... |  |
|  |  |  |  |  |  |  |  |  |  |  |  |  |  |  |  |  |  |  |  |  |  |  |  |  |  |  |  |  |  |  |  |  |  |  |  |  |  | 63 | 60 | 1.7 |  |  | 2: GRHL1\_1 ... |  |
|  |  |  |  |  |  |  |  |  |  |  |  |  |  |  |  |  |  |  |  |  |  |  |  |  |  |  |  |  |  |  |  |  |  |  |  |  |  | 64 | 48 | 20.5 |  |  | 3: pTH5882 ... |  |
|  |  |  |  |  |  |  |  |  |  |  |  |  |  |  |  |  |  |  |  |  |  |  |  |  |  |  |  |  |  |  |  |  |  |  |  |  |  | 65 | 27 | 12.9 |  |  | 2: Exex\_Cell\_FBgn0041156 ... |  |
|  |  |  |  |  |  |  |  |  |  |  |  |  |  |  |  |  |  |  |  |  |  |  |  |  |  |  |  |  |  |  |  |  |  |  |  |  |  | 66 | 37 | 2.6 |  |  | 16: TBX20\_5 ... |  |
|  |  |  |  |  |  |  |  |  |  |  |  |  |  |  |  |  |  |  |  |  |  |  |  |  |  |  |  |  |  |  |  |  |  |  |  |  |  | 67 | 112 | 88.6 |  |  | 757: Otp\_3496 ... |  |
|  |  |  |  |  |  |  |  |  |  |  |  |  |  |  |  |  |  |  |  |  |  |  |  |  |  |  |  |  |  |  |  |  |  |  |  |  |  | 68 | 42 | 2 |  |  | 56: Gata6\_3769 ... |  |
|  |  |  |  |  |  |  |  |  |  |  |  |  |  |  |  |  |  |  |  |  |  |  |  |  |  |  |  |  |  |  |  |  |  |  |  |  |  | 69 | 71 | 21.6 |  |  | 35: Hoxb6\_3428 ... |  |
|  |  |  |  |  |  |  |  |  |  |  |  |  |  |  |  |  |  |  |  |  |  |  |  |  |  |  |  |  |  |  |  |  |  |  |  |  |  | 70 | 27 | 29.2 |  |  |  |  |
|  |  |  |  |  |  |  |  |  |  |  |  |  |  |  |  |  |  |  |  |  |  |  |  |  |  |  |  |  |  |  |  |  |  |  |  |  |  | 71 | 44 | 5.6 |  |  | 2: PITX2\_si ... |  |
|  |  |  |  |  |  |  |  |  |  |  |  |  |  |  |  |  |  |  |  |  |  |  |  |  |  |  |  |  |  |  |  |  |  |  |  |  |  | 72 | 192 | 544.8 | 19: body wall musculature... | 41: 0hr muscle enriched... | 1098: pTH10808 ... | 15: EGL-5\_Larvae-L3-stage... |
|  |  |  |  |  |  |  |  |  |  |  |  |  |  |  |  |  |  |  |  |  |  |  |  |  |  |  |  |  |  |  |  |  |  |  |  |  |  | 73 | 63 | 114.6 |  |  | 72: pTH3467 ... | 2: MAB-5\_Embryos... |
|  |  |  |  |  |  |  |  |  |  |  |  |  |  |  |  |  |  |  |  |  |  |  |  |  |  |  |  |  |  |  |  |  |  |  |  |  |  | 74 | 144 | 18.9 |  |  | 46: Hoxd11\_3873 ... |  |
|  |  |  |  |  |  |  |  |  |  |  |  |  |  |  |  |  |  |  |  |  |  |  |  |  |  |  |  |  |  |  |  |  |  |  |  |  |  | 75 | 192 | 9.9 |  |  | 2: Hoxd9\_3 ... | 1: HAM-1\_Fed-L1-stage-larvae... |
|  |  |  |  |  |  |  |  |  |  |  |  |  |  |  |  |  |  |  |  |  |  |  |  |  |  |  |  |  |  |  |  |  |  |  |  |  |  | 76 | 115 | 0.1 |  |  | 25: bin\_FlyReg\_FBgn0045759 ... |  |
|  |  |  |  |  |  |  |  |  |  |  |  |  |  |  |  |  |  |  |  |  |  |  |  |  |  |  |  |  |  |  |  |  |  |  |  |  |  | 77 | 24 | 39.6 |  |  | 6: MA0579.1 ... |  |
|  |  |  |  |  |  |  |  |  |  |  |  |  |  |  |  |  |  |  |  |  |  |  |  |  |  |  |  |  |  |  |  |  |  |  |  |  |  | 78 | 39 | 2.2 |  |  | 6: pTH3997 ... |  |
|  |  |  |  |  |  |  |  |  |  |  |  |  |  |  |  |  |  |  |  |  |  |  |  |  |  |  |  |  |  |  |  |  |  |  |  |  |  | 79 | 80 | 2.4 |  | 7: WBPaper00025032:cluster 33... | 2: OTX2\_1 ... |  |
|  |  |  |  |  |  |  |  |  |  |  |  |  |  |  |  |  |  |  |  |  |  |  |  |  |  |  |  |  |  |  |  |  |  |  |  |  |  | 80 | 91 | 94.9 |  | 9: [cgc5767]:expression class MD... | 10: DLX2\_f1 ... | 4: NHR-77\_Larvae-L4-stage... |
|  |  |  |  |  |  |  |  |  |  |  |  |  |  |  |  |  |  |  |  |  |  |  |  |  |  |  |  |  |  |  |  |  |  |  |  |  |  | 81 | 169 | 4.7 |  |  | 229: MCR\_f1 ... | 1: PHA-4\_Larvae-L4-stage... |
|  |  |  |  |  |  |  |  |  |  |  |  |  |  |  |  |  |  |  |  |  |  |  |  |  |  |  |  |  |  |  |  |  |  |  |  |  |  | 82 | 74 | 34.5 |  |  | 2: SPDEF\_5 ... |  |
|  |  |  |  |  |  |  |  |  |  |  |  |  |  |  |  |  |  |  |  |  |  |  |  |  |  |  |  |  |  |  |  |  |  |  |  |  |  | 83 | 61 | 81 |  |  | 276: pTH6636 ... | 4: NHR-77\_Larvae-L4-stage... |
|  |  |  |  |  |  |  |  |  |  |  |  |  |  |  |  |  |  |  |  |  |  |  |  |  |  |  |  |  |  |  |  |  |  |  |  |  |  | 84 | 87 | 130.5 |  | 4: excretory-cell larva selectively enriched... | 136: OTX1\_f1 ... |  |
|  |  |  |  |  |  |  |  |  |  |  |  |  |  |  |  |  |  |  |  |  |  |  |  |  |  |  |  |  |  |  |  |  |  |  |  |  |  | 85 | 117 | 119.4 |  |  | 30: Eip93F\_SANGER\_10\_FBgn0013948 ... | 3: ELT-3\_Embryos... |
|  |  |  |  |  |  |  |  |  |  |  |  |  |  |  |  |  |  |  |  |  |  |  |  |  |  |  |  |  |  |  |  |  |  |  |  |  |  | 86 | 133 | 5.6 |  |  | 110: pTH6497 ... | 1: DPL-1\_Larvae-L4-stage... |
|  |  |  |  |  |  |  |  |  |  |  |  |  |  |  |  |  |  |  |  |  |  |  |  |  |  |  |  |  |  |  |  |  |  |  |  |  |  | 87 | 29 | 3.9 |  |  | 6: HXA9\_f1 ... |  |
|  |  |  |  |  |  |  |  |  |  |  |  |  |  |  |  |  |  |  |  |  |  |  |  |  |  |  |  |  |  |  |  |  |  |  |  |  |  | 88 | 29 | 96.1 |  |  | 29: MA0456.1 ... |  |
|  |  |  |  |  |  |  |  |  |  |  |  |  |  |  |  |  |  |  |  |  |  |  |  |  |  |  |  |  |  |  |  |  |  |  |  |  |  | 89 | 17 | 0.5 |  |  | 4: TFAP4\_2 ... |  |
|  |  |  |  |  |  |  |  |  |  |  |  |  |  |  |  |  |  |  |  |  |  |  |  |  |  |  |  |  |  |  |  |  |  |  |  |  |  | 90 | 111 | 1 |  |  | 25: COT1\_si ... |  |
|  |  |  |  |  |  |  |  |  |  |  |  |  |  |  |  |  |  |  |  |  |  |  |  |  |  |  |  |  |  |  |  |  |  |  |  |  |  | 91 | 33 | 129.2 |  |  | 76: GM12878\_EGR1\_HudsonAlpha ... |  |
|  |  |  |  |  |  |  |  |  |  |  |  |  |  |  |  |  |  |  |  |  |  |  |  |  |  |  |  |  |  |  |  |  |  |  |  |  |  | 92 | 17 | 27.7 |  |  | 4: V$CDP\_01 ... | 1: F23F12.9\_Embryos... |
|  |  |  |  |  |  |  |  |  |  |  |  |  |  |  |  |  |  |  |  |  |  |  |  |  |  |  |  |  |  |  |  |  |  |  |  |  |  | 93 | 37 | 84.9 |  |  | 11: V$CEBP\_01 ... |  |
|  |  |  |  |  |  |  |  |  |  |  |  |  |  |  |  |  |  |  |  |  |  |  |  |  |  |  |  |  |  |  |  |  |  |  |  |  |  | 94 | 41 | 5.7 |  |  | 5: pTH9059 ... |  |
|  |  |  |  |  |  |  |  |  |  |  |  |  |  |  |  |  |  |  |  |  |  |  |  |  |  |  |  |  |  |  |  |  |  |  |  |  |  | 95 | 192 | 129.6 | 4: body wall musculature... | 26: 0hr muscle enriched... | 1621: pTH10808 ... | 9: EGL-5\_Larvae-L3-stage... |
|  |  |  |  |  |  |  |  |  |  |  |  |  |  |  |  |  |  |  |  |  |  |  |  |  |  |  |  |  |  |  |  |  |  |  |  |  |  | 96 | 357 | 147.8 |  | 81: hypodermis embryo enriched... | 1635: So\_SOLEXA\_FBgn0003460 ... | 12: NHR-28\_Larvae-L4-stage... |
|  |  |  |  |  |  |  |  |  |  |  |  |  |  |  |  |  |  |  |  |  |  |  |  |  |  |  |  |  |  |  |  |  |  |  |  |  |  | 97 | 23 | 0.4 |  |  | 62: V$RFX1\_01 ... |  |
|  |  |  |  |  |  |  |  |  |  |  |  |  |  |  |  |  |  |  |  |  |  |  |  |  |  |  |  |  |  |  |  |  |  |  |  |  |  | 98 | 115 | 473.6 | 5: OLLsoR... | 40: hypodermis embryo selectively enriched... | 1253: pTH3811 ... | 23: NHR-23\_Larvae-L3-stage... |
|  |  |  |  |  |  |  |  |  |  |  |  |  |  |  |  |  |  |  |  |  |  |  |  |  |  |  |  |  |  |  |  |  |  |  |  |  |  | 99 | 93 | 47.1 |  |  | 28: ftz-f1\_FlyReg\_FBgn0001078 ... |  |
|  |  |  |  |  |  |  |  |  |  |  |  |  |  |  |  |  |  |  |  |  |  |  |  |  |  |  |  |  |  |  |  |  |  |  |  |  |  | 100 | 41 | 7.9 |  |  | 1: V$AP4\_Q6 ... |  |
|  |  |  |  |  |  |  |  |  |  |  |  |  |  |  |  |  |  |  |  |  |  |  |  |  |  |  |  |  |  |  |  |  |  |  |  |  |  | 101 | 37 | 31.1 |  |  | 50: V$E47\_01 ... |  |
|  |  |  |  |  |  |  |  |  |  |  |  |  |  |  |  |  |  |  |  |  |  |  |  |  |  |  |  |  |  |  |  |  |  |  |  |  |  | 102 | 231 | 5.4 |  |  | 127: GM12878\_ELF1\_HudsonAlpha ... |  |
|  |  |  |  |  |  |  |  |  |  |  |  |  |  |  |  |  |  |  |  |  |  |  |  |  |  |  |  |  |  |  |  |  |  |  |  |  |  | 103 | 41 | 30.4 |  |  | 11: Mv104 ... | 1: LIN-35\_Young-adult... |
|  |  |  |  |  |  |  |  |  |  |  |  |  |  |  |  |  |  |  |  |  |  |  |  |  |  |  |  |  |  |  |  |  |  |  |  |  |  | 104 | 46 | 0.4 |  |  | 1: ftz\_FlyReg\_FBgn0001077 ... |  |
|  |  |  |  |  |  |  |  |  |  |  |  |  |  |  |  |  |  |  |  |  |  |  |  |  |  |  |  |  |  |  |  |  |  |  |  |  |  | 105 | 56 | 129.1 | 7: DB neuron... | 5: all-neurons larva enriched... | 1389: pTH9957 ... |  |
|  |  |  |  |  |  |  |  |  |  |  |  |  |  |  |  |  |  |  |  |  |  |  |  |  |  |  |  |  |  |  |  |  |  |  |  |  |  | 106 | 65 | 53.3 |  |  | 52: Cutl1\_3494 ... |  |
|  |  |  |  |  |  |  |  |  |  |  |  |  |  |  |  |  |  |  |  |  |  |  |  |  |  |  |  |  |  |  |  |  |  |  |  |  |  | 107 | 60 | 5.8 |  |  | 8: Pou2f2\_1 ... | 4: NHR-77\_Larvae-L2-stage... |
|  |  |  |  |  |  |  |  |  |  |  |  |  |  |  |  |  |  |  |  |  |  |  |  |  |  |  |  |  |  |  |  |  |  |  |  |  |  | 108 | 51 | 1.8 |  |  | 2: pTH9049 ... |  |
|  |  |  |  |  |  |  |  |  |  |  |  |  |  |  |  |  |  |  |  |  |  |  |  |  |  |  |  |  |  |  |  |  |  |  |  |  |  | 109 | 242 | 34.1 |  |  | 22: pTH9163 ... | 3: AHA-1\_Larvae-L4-stage... |
|  |  |  |  |  |  |  |  |  |  |  |  |  |  |  |  |  |  |  |  |  |  |  |  |  |  |  |  |  |  |  |  |  |  |  |  |  |  | 110 | 104 | 41 |  |  | 103: SHOX2\_1 ... |  |
|  |  |  |  |  |  |  |  |  |  |  |  |  |  |  |  |  |  |  |  |  |  |  |  |  |  |  |  |  |  |  |  |  |  |  |  |  |  | 111 | 68 | 20.8 |  |  | 123: ems\_FlyReg\_FBgn0000576 ... |  |
|  |  |  |  |  |  |  |  |  |  |  |  |  |  |  |  |  |  |  |  |  |  |  |  |  |  |  |  |  |  |  |  |  |  |  |  |  |  | 112 | 67 | 0.7 |  |  | 1: Sox1\_4 ... |  |
|  |  |  |  |  |  |  |  |  |  |  |  |  |  |  |  |  |  |  |  |  |  |  |  |  |  |  |  |  |  |  |  |  |  |  |  |  |  | 113 | 73 | 28.5 |  |  | 14: V$HLF\_01 ... |  |
|  |  |  |  |  |  |  |  |  |  |  |  |  |  |  |  |  |  |  |  |  |  |  |  |  |  |  |  |  |  |  |  |  |  |  |  |  |  | 114 | 70 | 2.5 |  |  | 7: MA0222.1 ... |  |
|  |  |  |  |  |  |  |  |  |  |  |  |  |  |  |  |  |  |  |  |  |  |  |  |  |  |  |  |  |  |  |  |  |  |  |  |  |  | 115 | 56 | 312.1 |  |  | 272: I$E74A\_01 ... | 1: DPL-1\_Larvae-L4-stage... |
|  |  |  |  |  |  |  |  |  |  |  |  |  |  |  |  |  |  |  |  |  |  |  |  |  |  |  |  |  |  |  |  |  |  |  |  |  |  | 116 | 34 | 39.9 |  |  | 1: pTH9478 ... | 1: ALY-2\_Larvae-L2-stage... |
|  |  |  |  |  |  |  |  |  |  |  |  |  |  |  |  |  |  |  |  |  |  |  |  |  |  |  |  |  |  |  |  |  |  |  |  |  |  | 117 | 279 | 12.9 |  | 1: WBPaper00025032:cluster 69... | 92: EGR1\_f2 ... | 112: ZTF-11\_Embryos... |
|  |  |  |  |  |  |  |  |  |  |  |  |  |  |  |  |  |  |  |  |  |  |  |  |  |  |  |  |  |  |  |  |  |  |  |  |  |  | 118 | 227 | 302 |  | 22: P.lumniescens 24hr upregulated RNAseq... | 875: MA0016.1 ... | 3: PHA-4\_Larvae-L2-stage... |
|  |  |  |  |  |  |  |  |  |  |  |  |  |  |  |  |  |  |  |  |  |  |  |  |  |  |  |  |  |  |  |  |  |  |  |  |  |  | 119 | 21 | 0.5 |  |  | 3: pTH6482 ... |  |
|  |  |  |  |  |  |  |  |  |  |  |  |  |  |  |  |  |  |  |  |  |  |  |  |  |  |  |  |  |  |  |  |  |  |  |  |  |  | 120 | 20 | 0.9 |  |  | 3: NR2C1\_si ... |  |
|  |  |  |  |  |  |  |  |  |  |  |  |  |  |  |  |  |  |  |  |  |  |  |  |  |  |  |  |  |  |  |  |  |  |  |  |  |  | 121 | 40 | 4.9 |  |  | 6: V$CREB\_Q4 ... |  |
|  |  |  |  |  |  |  |  |  |  |  |  |  |  |  |  |  |  |  |  |  |  |  |  |  |  |  |  |  |  |  |  |  |  |  |  |  |  | 122 | 65 | 182.2 |  | 4: [cgc5767]:expression class M... | 6: pTH8982 ... | 68: F23B12.7\_Young-adult... |
|  |  |  |  |  |  |  |  |  |  |  |  |  |  |  |  |  |  |  |  |  |  |  |  |  |  |  |  |  |  |  |  |  |  |  |  |  |  | 123 | 27 | 2.8 |  |  | 24: FOXO3\_1 ... |  |
|  |  |  |  |  |  |  |  |  |  |  |  |  |  |  |  |  |  |  |  |  |  |  |  |  |  |  |  |  |  |  |  |  |  |  |  |  |  | 124 | 77 | 31.6 |  |  | 9: pTH5018 ... |  |
|  |  |  |  |  |  |  |  |  |  |  |  |  |  |  |  |  |  |  |  |  |  |  |  |  |  |  |  |  |  |  |  |  |  |  |  |  |  | 125 | 19 | 25.8 |  |  | 2: NR4A2\_2 ... |  |
|  |  |  |  |  |  |  |  |  |  |  |  |  |  |  |  |  |  |  |  |  |  |  |  |  |  |  |  |  |  |  |  |  |  |  |  |  |  | 126 | 435 | 0.4 |  |  | 4: Osr2\_1727 ... |  |
|  |  |  |  |  |  |  |  |  |  |  |  |  |  |  |  |  |  |  |  |  |  |  |  |  |  |  |  |  |  |  |  |  |  |  |  |  |  | 127 | 58 | 2.9 |  |  |  |  |
|  |  |  |  |  |  |  |  |  |  |  |  |  |  |  |  |  |  |  |  |  |  |  |  |  |  |  |  |  |  |  |  |  |  |  |  |  |  | 128 | 26 | 25.6 |  |  | 1: V$AREB6\_01 ... |  |
|  |  |  |  |  |  |  |  |  |  |  |  |  |  |  |  |  |  |  |  |  |  |  |  |  |  |  |  |  |  |  |  |  |  |  |  |  |  | 129 | 208 | 3.7 |  | 9: intestine embryo enriched... | 45: Mw142 ... | 8: FOS-1\_Larvae-L2-stage... |
|  |  |  |  |  |  |  |  |  |  |  |  |  |  |  |  |  |  |  |  |  |  |  |  |  |  |  |  |  |  |  |  |  |  |  |  |  |  | 130 | 50 | 135.8 |  |  | 37: ftz-f1\_FlyReg\_FBgn0001078 ... | 1: GEI-11\_Larvae-L3-stage... |
|  |  |  |  |  |  |  |  |  |  |  |  |  |  |  |  |  |  |  |  |  |  |  |  |  |  |  |  |  |  |  |  |  |  |  |  |  |  | 131 | 259 | 8.1 | 1: germ line... | 59: germline-precursors embryo enriched... | 15: pTH10810 ... | 4: EFL-1\_Young-adult... |
|  |  |  |  |  |  |  |  |  |  |  |  |  |  |  |  |  |  |  |  |  |  |  |  |  |  |  |  |  |  |  |  |  |  |  |  |  |  | 132 | 59 | 245.1 |  | 1: mir-243 down regulated... | 1: srp\_SANGER\_5\_FBgn0003507 ... | 5: W03F9.2\_L4-Young-Adult-stage-larvae... |
|  |  |  |  |  |  |  |  |  |  |  |  |  |  |  |  |  |  |  |  |  |  |  |  |  |  |  |  |  |  |  |  |  |  |  |  |  |  | 133 | 29 | 3.3 |  |  | 2: HXA10\_f1 ... |  |
|  |  |  |  |  |  |  |  |  |  |  |  |  |  |  |  |  |  |  |  |  |  |  |  |  |  |  |  |  |  |  |  |  |  |  |  |  |  | 134 | 57 | 5.2 |  |  | 1: PAX8\_f1 ... |  |
|  |  |  |  |  |  |  |  |  |  |  |  |  |  |  |  |  |  |  |  |  |  |  |  |  |  |  |  |  |  |  |  |  |  |  |  |  |  | 135 | 38 | 7.8 |  |  | 1: CG8765\_SANGER\_5\_FBgn0036900 ... | 6: DPL-1\_Young-adult... |
|  |  |  |  |  |  |  |  |  |  |  |  |  |  |  |  |  |  |  |  |  |  |  |  |  |  |  |  |  |  |  |  |  |  |  |  |  |  | 136 | 44 | 0.9 |  |  | 1: pTH9326 ... |  |
|  |  |  |  |  |  |  |  |  |  |  |  |  |  |  |  |  |  |  |  |  |  |  |  |  |  |  |  |  |  |  |  |  |  |  |  |  |  | 137 | 61 | 9.6 |  | 12: germline-precursors embryo enriched... | 1: Zfp161\_2858 ... | 2: EFL-1\_Young-adult... |
|  |  |  |  |  |  |  |  |  |  |  |  |  |  |  |  |  |  |  |  |  |  |  |  |  |  |  |  |  |  |  |  |  |  |  |  |  |  | 138 | 70 | 28.1 |  |  | 32: EVX1\_1 ... |  |
|  |  |  |  |  |  |  |  |  |  |  |  |  |  |  |  |  |  |  |  |  |  |  |  |  |  |  |  |  |  |  |  |  |  |  |  |  |  | 139 | 222 | 43.7 |  | 1: AFD vs AWB upregulated... | 35: En2\_1 ... |  |
|  |  |  |  |  |  |  |  |  |  |  |  |  |  |  |  |  |  |  |  |  |  |  |  |  |  |  |  |  |  |  |  |  |  |  |  |  |  | 140 | 77 | 173.8 |  | 10: CE B.thuringiensis-DB27 regulated... | 56: pTH8997 ... | 87: EFL-1\_Fed-L1-stage-larvae... |
|  |  |  |  |  |  |  |  |  |  |  |  |  |  |  |  |  |  |  |  |  |  |  |  |  |  |  |  |  |  |  |  |  |  |  |  |  |  | 141 | 108 | 242.6 |  | 18: FBF-1 Associated... | 22: Mw138 ... | 49: EFL-1\_Larvae-L1-stage... |
|  |  |  |  |  |  |  |  |  |  |  |  |  |  |  |  |  |  |  |  |  |  |  |  |  |  |  |  |  |  |  |  |  |  |  |  |  |  | 142 | 34 | 0.2 |  |  | 16: RAX\_1 ... |  |
|  |  |  |  |  |  |  |  |  |  |  |  |  |  |  |  |  |  |  |  |  |  |  |  |  |  |  |  |  |  |  |  |  |  |  |  |  |  | 143 | 27 | 0.6 |  |  |  |  |
|  |  |  |  |  |  |  |  |  |  |  |  |  |  |  |  |  |  |  |  |  |  |  |  |  |  |  |  |  |  |  |  |  |  |  |  |  |  | 144 | 75 | 10.1 |  |  | 12: EVX1\_1 ... | 1: HAM-1\_Fed-L1-stage-larvae... |
|  |  |  |  |  |  |  |  |  |  |  |  |  |  |  |  |  |  |  |  |  |  |  |  |  |  |  |  |  |  |  |  |  |  |  |  |  |  | 145 | 102 | 0.6 |  |  | 23: Hey\_SANGER\_5\_FBgn0027788 ... |  |
|  |  |  |  |  |  |  |  |  |  |  |  |  |  |  |  |  |  |  |  |  |  |  |  |  |  |  |  |  |  |  |  |  |  |  |  |  |  | 146 | 193 | 104.5 |  |  | 17: CRX\_si ... |  |
|  |  |  |  |  |  |  |  |  |  |  |  |  |  |  |  |  |  |  |  |  |  |  |  |  |  |  |  |  |  |  |  |  |  |  |  |  |  | 147 | 30 | 0.5 |  |  | 5: Hoxd9\_2 ... |  |
|  |  |  |  |  |  |  |  |  |  |  |  |  |  |  |  |  |  |  |  |  |  |  |  |  |  |  |  |  |  |  |  |  |  |  |  |  |  | 148 | 67 | 214.6 |  |  |  | 17: LSY-2\_Embryos... |
|  |  |  |  |  |  |  |  |  |  |  |  |  |  |  |  |  |  |  |  |  |  |  |  |  |  |  |  |  |  |  |  |  |  |  |  |  |  | 149 | 25 | 4.4 |  |  | 11: MA0174.1 ... |  |
|  |  |  |  |  |  |  |  |  |  |  |  |  |  |  |  |  |  |  |  |  |  |  |  |  |  |  |  |  |  |  |  |  |  |  |  |  |  | 150 | 33 | 0.4 |  |  |  |  |
|  |  |  |  |  |  |  |  |  |  |  |  |  |  |  |  |  |  |  |  |  |  |  |  |  |  |  |  |  |  |  |  |  |  |  |  |  |  | 151 | 80 | 3.5 |  |  | 8: pTH9199 ... |  |
|  |  |  |  |  |  |  |  |  |  |  |  |  |  |  |  |  |  |  |  |  |  |  |  |  |  |  |  |  |  |  |  |  |  |  |  |  |  | 152 | 34 | 24.6 |  |  | 11: HIF1A\_si ... |  |
|  |  |  |  |  |  |  |  |  |  |  |  |  |  |  |  |  |  |  |  |  |  |  |  |  |  |  |  |  |  |  |  |  |  |  |  |  |  | 153 | 86 | 323.2 |  | 3: [cgc5767]:expression class MD... | 13: ONECUT2\_1 ... | 44: EOR-1\_Larvae-L3-stage... |
|  |  |  |  |  |  |  |  |  |  |  |  |  |  |  |  |  |  |  |  |  |  |  |  |  |  |  |  |  |  |  |  |  |  |  |  |  |  | 154 | 34 | 392 |  | 7: [cgc5428]:L1 muscle enriched... | 24: pTH10030 ... | 98: EFL-1\_Larvae-L1-stage... |
|  |  |  |  |  |  |  |  |  |  |  |  |  |  |  |  |  |  |  |  |  |  |  |  |  |  |  |  |  |  |  |  |  |  |  |  |  |  | 155 | 52 | 125.2 |  | 10: hypodermis larva selectively enriched... | 134: pTH2283 ... | 8: NHR-23\_Larvae-L3-stage... |
|  |  |  |  |  |  |  |  |  |  |  |  |  |  |  |  |  |  |  |  |  |  |  |  |  |  |  |  |  |  |  |  |  |  |  |  |  |  | 156 | 90 | 342.2 |  | 1: dopaminergic-neurons larva enriched... | 8: pTH3220 ... | 27: CES-1\_Embryos... |
|  |  |  |  |  |  |  |  |  |  |  |  |  |  |  |  |  |  |  |  |  |  |  |  |  |  |  |  |  |  |  |  |  |  |  |  |  |  | 157 | 147 | 407.9 |  | 14: Larva Pan Neuronal Enriched... | 1143: MA0543.1 ... | 82: CES-1\_Embryos... |
|  |  |  |  |  |  |  |  |  |  |  |  |  |  |  |  |  |  |  |  |  |  |  |  |  |  |  |  |  |  |  |  |  |  |  |  |  |  | 158 | 96 | 281 |  | 7: 0hr muscle enriched... | 501: V$SP1\_Q6 ... | 32: CES-1\_Embryos... |
|  |  |  |  |  |  |  |  |  |  |  |  |  |  |  |  |  |  |  |  |  |  |  |  |  |  |  |  |  |  |  |  |  |  |  |  |  |  | 159 | 88 | 0.3 |  |  | 10: pTH10816 ... |  |
|  |  |  |  |  |  |  |  |  |  |  |  |  |  |  |  |  |  |  |  |  |  |  |  |  |  |  |  |  |  |  |  |  |  |  |  |  |  | 160 | 79 | 3.5 |  |  | 16: PITX1\_3 ... |  |
|  |  |  |  |  |  |  |  |  |  |  |  |  |  |  |  |  |  |  |  |  |  |  |  |  |  |  |  |  |  |  |  |  |  |  |  |  |  | 161 | 65 | 551.4 |  | 13: 0hr muscle enriched... | 1013: MA0039.2 ... | 11: ZAG-1\_Larvae-L2-stage... |
|  |  |  |  |  |  |  |  |  |  |  |  |  |  |  |  |  |  |  |  |  |  |  |  |  |  |  |  |  |  |  |  |  |  |  |  |  |  | 162 | 47 | 2.5 |  |  | 9: RORA\_f1 ... |  |
|  |  |  |  |  |  |  |  |  |  |  |  |  |  |  |  |  |  |  |  |  |  |  |  |  |  |  |  |  |  |  |  |  |  |  |  |  |  | 163 | 27 | 1.1 |  |  | 5: tgo\_sim\_SANGER\_5\_FBgn0015014 ... |  |
|  |  |  |  |  |  |  |  |  |  |  |  |  |  |  |  |  |  |  |  |  |  |  |  |  |  |  |  |  |  |  |  |  |  |  |  |  |  | 164 | 23 | 2.3 |  |  | 8: Isl2\_3430 ... |  |
|  |  |  |  |  |  |  |  |  |  |  |  |  |  |  |  |  |  |  |  |  |  |  |  |  |  |  |  |  |  |  |  |  |  |  |  |  |  | 165 | 125 | 0.2 |  |  | 40: pTH10038 ... | 3: NHR-28\_Larvae-L4-stage... |
|  |  |  |  |  |  |  |  |  |  |  |  |  |  |  |  |  |  |  |  |  |  |  |  |  |  |  |  |  |  |  |  |  |  |  |  |  |  | 166 | 38 | 0.6 |  |  | 3: tll\_FlyReg\_FBgn0003720 ... |  |
|  |  |  |  |  |  |  |  |  |  |  |  |  |  |  |  |  |  |  |  |  |  |  |  |  |  |  |  |  |  |  |  |  |  |  |  |  |  | 167 | 51 | 0.6 |  |  | 4: SRF\_do ... |  |
|  |  |  |  |  |  |  |  |  |  |  |  |  |  |  |  |  |  |  |  |  |  |  |  |  |  |  |  |  |  |  |  |  |  |  |  |  |  | 168 | 34 | 9.9 |  |  | 1: MYB\_f1 ... |  |
|  |  |  |  |  |  |  |  |  |  |  |  |  |  |  |  |  |  |  |  |  |  |  |  |  |  |  |  |  |  |  |  |  |  |  |  |  |  | 169 | 126 | 262 |  | 38: L3 enriched... | 431: pTH10624 ... | 112: EFL-1\_Fed-L1-stage-larvae... |
|  |  |  |  |  |  |  |  |  |  |  |  |  |  |  |  |  |  |  |  |  |  |  |  |  |  |  |  |  |  |  |  |  |  |  |  |  |  | 170 | 50 | 396.4 |  | 11: E.faecalis 24hr downregulated RNAseq... | 87: ONECUT1\_1 ... | 93: EFL-1\_Fed-L1-stage-larvae... |
|  |  |  |  |  |  |  |  |  |  |  |  |  |  |  |  |  |  |  |  |  |  |  |  |  |  |  |  |  |  |  |  |  |  |  |  |  |  | 171 | 130 | 172.6 |  | 27: CE B.thuringiensis-DB27 regulated... | 87: pTH7876 ... | 64: EFL-1\_Fed-L1-stage-larvae... |
|  |  |  |  |  |  |  |  |  |  |  |  |  |  |  |  |  |  |  |  |  |  |  |  |  |  |  |  |  |  |  |  |  |  |  |  |  |  | 172 | 14 | 6.6 |  |  |  |  |
|  |  |  |  |  |  |  |  |  |  |  |  |  |  |  |  |  |  |  |  |  |  |  |  |  |  |  |  |  |  |  |  |  |  |  |  |  |  | 173 | 23 | 3.1 |  | 1: hcf-1nc sir-2.1down daf-2down... |  |  |
|  |  |  |  |  |  |  |  |  |  |  |  |  |  |  |  |  |  |  |  |  |  |  |  |  |  |  |  |  |  |  |  |  |  |  |  |  |  | 174 | 70 | 128.1 |  | 16: CE B.thuringiensis-DB27 regulated... | 518: FOXC2\_2 ... | 29: EFL-1\_Larvae-L1-stage... |
|  |  |  |  |  |  |  |  |  |  |  |  |  |  |  |  |  |  |  |  |  |  |  |  |  |  |  |  |  |  |  |  |  |  |  |  |  |  | 175 | 67 | 132.1 |  |  | 8: V$MEF2\_01 ... | 20: NFYA-1\_Larvae-L3-stage... |
|  |  |  |  |  |  |  |  |  |  |  |  |  |  |  |  |  |  |  |  |  |  |  |  |  |  |  |  |  |  |  |  |  |  |  |  |  |  | 176 | 32 | 68.6 |  |  | 4: sv\_SOLEXA\_5\_FBgn0005561 ... |  |
|  |  |  |  |  |  |  |  |  |  |  |  |  |  |  |  |  |  |  |  |  |  |  |  |  |  |  |  |  |  |  |  |  |  |  |  |  |  | 177 | 44 | 126.3 |  |  | 12: ELK1\_f1 ... |  |
|  |  |  |  |  |  |  |  |  |  |  |  |  |  |  |  |  |  |  |  |  |  |  |  |  |  |  |  |  |  |  |  |  |  |  |  |  |  | 178 | 18 | 8.6 |  |  |  |  |
|  |  |  |  |  |  |  |  |  |  |  |  |  |  |  |  |  |  |  |  |  |  |  |  |  |  |  |  |  |  |  |  |  |  |  |  |  |  | 179 | 43 | 110 |  |  | 7: SPDEF\_6 ... | 1: EGL-5\_Larvae-L3-stage... |
|  |  |  |  |  |  |  |  |  |  |  |  |  |  |  |  |  |  |  |  |  |  |  |  |  |  |  |  |  |  |  |  |  |  |  |  |  |  | 180 | 316 | 18.4 |  |  | 1439: Plagl1\_0972 ... | 31: PES-1\_Larvae-L4-stage... |
|  |  |  |  |  |  |  |  |  |  |  |  |  |  |  |  |  |  |  |  |  |  |  |  |  |  |  |  |  |  |  |  |  |  |  |  |  |  | 181 | 62 | 29.2 |  |  | 105: pTH9089 ... | 4: LIN-35\_Starved-L1-stage-larvae... |
|  |  |  |  |  |  |  |  |  |  |  |  |  |  |  |  |  |  |  |  |  |  |  |  |  |  |  |  |  |  |  |  |  |  |  |  |  |  | 182 | 29 | 12.6 |  |  | 8: pTH9173 ... |  |
|  |  |  |  |  |  |  |  |  |  |  |  |  |  |  |  |  |  |  |  |  |  |  |  |  |  |  |  |  |  |  |  |  |  |  |  |  |  | 183 | 167 | 8.9 |  |  | 47: V$CMYB\_01 ... | 5: ZTF-11\_Embryos... |
|  |  |  |  |  |  |  |  |  |  |  |  |  |  |  |  |  |  |  |  |  |  |  |  |  |  |  |  |  |  |  |  |  |  |  |  |  |  | 184 | 172 | 0.3 |  |  | 110: Hey\_SANGER\_5\_FBgn0027788 ... | 1: EGL-5\_Larvae-L3-stage... |
|  |  |  |  |  |  |  |  |  |  |  |  |  |  |  |  |  |  |  |  |  |  |  |  |  |  |  |  |  |  |  |  |  |  |  |  |  |  | 185 | 59 | 0.6 |  |  | 2: SPDEF\_5 ... |  |
|  |  |  |  |  |  |  |  |  |  |  |  |  |  |  |  |  |  |  |  |  |  |  |  |  |  |  |  |  |  |  |  |  |  |  |  |  |  | 186 | 50 | 0.7 |  |  |  |  |
|  |  |  |  |  |  |  |  |  |  |  |  |  |  |  |  |  |  |  |  |  |  |  |  |  |  |  |  |  |  |  |  |  |  |  |  |  |  | 187 | 93 | 0.8 |  |  | 1: Mv75 ... |  |
|  |  |  |  |  |  |  |  |  |  |  |  |  |  |  |  |  |  |  |  |  |  |  |  |  |  |  |  |  |  |  |  |  |  |  |  |  |  | 188 | 29 | 14.8 |  |  | 5: pTH6201 ... |  |
|  |  |  |  |  |  |  |  |  |  |  |  |  |  |  |  |  |  |  |  |  |  |  |  |  |  |  |  |  |  |  |  |  |  |  |  |  |  | 189 | 240 | 18.4 |  | 1: AFD vs AWB upregulated... | 54: OTX1\_2 ... |  |
|  |  |  |  |  |  |  |  |  |  |  |  |  |  |  |  |  |  |  |  |  |  |  |  |  |  |  |  |  |  |  |  |  |  |  |  |  |  | 190 | 33 | 4.9 |  |  | 1: ETV4\_f1 ... |  |
|  |  |  |  |  |  |  |  |  |  |  |  |  |  |  |  |  |  |  |  |  |  |  |  |  |  |  |  |  |  |  |  |  |  |  |  |  |  | 191 | 35 | 6.8 |  | 2: crh-1null up regulated... | 3: pTH9132 ... |  |
|  |  |  |  |  |  |  |  |  |  |  |  |  |  |  |  |  |  |  |  |  |  |  |  |  |  |  |  |  |  |  |  |  |  |  |  |  |  | 192 | 38 | 1.5 |  |  | 1: ftz-f1\_FlyReg\_FBgn0001078 ... |  |
|  |  |  |  |  |  |  |  |  |  |  |  |  |  |  |  |  |  |  |  |  |  |  |  |  |  |  |  |  |  |  |  |  |  |  |  |  |  | 193 | 17 | 0.7 |  |  | 2: NR4A2\_si ... |  |
|  |  |  |  |  |  |  |  |  |  |  |  |  |  |  |  |  |  |  |  |  |  |  |  |  |  |  |  |  |  |  |  |  |  |  |  |  |  | 194 | 35 | 8.8 |  |  | 13: MA0536.1 ... | 6: MAB-5\_Embryos... |
|  |  |  |  |  |  |  |  |  |  |  |  |  |  |  |  |  |  |  |  |  |  |  |  |  |  |  |  |  |  |  |  |  |  |  |  |  |  | 195 | 15 | 30.3 |  |  | 8: pTH10777 ... | 1: NHR-116\_Larvae-L4-stage... |
|  |  |  |  |  |  |  |  |  |  |  |  |  |  |  |  |  |  |  |  |  |  |  |  |  |  |  |  |  |  |  |  |  |  |  |  |  |  | 196 | 20 | 1.3 |  |  |  |  |
|  |  |  |  |  |  |  |  |  |  |  |  |  |  |  |  |  |  |  |  |  |  |  |  |  |  |  |  |  |  |  |  |  |  |  |  |  |  | 197 | 14 | 14.4 |  |  | 1: pTH3037 ... |  |
|  |  |  |  |  |  |  |  |  |  |  |  |  |  |  |  |  |  |  |  |  |  |  |  |  |  |  |  |  |  |  |  |  |  |  |  |  |  | 198 | 52 | 16.4 |  | 32: [cgc5767]:expression class SET... | 35: pTH8982 ... |  |
|  |  |  |  |  |  |  |  |  |  |  |  |  |  |  |  |  |  |  |  |  |  |  |  |  |  |  |  |  |  |  |  |  |  |  |  |  |  | 199 | 34 | 44.2 |  |  | 4: MA0173.1 ... |  |
|  |  |  |  |  |  |  |  |  |  |  |  |  |  |  |  |  |  |  |  |  |  |  |  |  |  |  |  |  |  |  |  |  |  |  |  |  |  | 200 | 42 | 6.8 |  |  | 5: pTH9285 ... |  |
|  |  |  |  |  |  |  |  |  |  |  |  |  |  |  |  |  |  |  |  |  |  |  |  |  |  |  |  |  |  |  |  |  |  |  |  |  |  | 201 | 30 | 37.2 |  |  | 1: Scr\_Cell\_FBgn0003339 ... |  |
|  |  |  |  |  |  |  |  |  |  |  |  |  |  |  |  |  |  |  |  |  |  |  |  |  |  |  |  |  |  |  |  |  |  |  |  |  |  | 202 | 83 | 0.3 |  |  | 61: SOX10\_1 ... | 5: ELT-1\_Larvae-L3-stage... |
|  |  |  |  |  |  |  |  |  |  |  |  |  |  |  |  |  |  |  |  |  |  |  |  |  |  |  |  |  |  |  |  |  |  |  |  |  |  | 203 | 41 | 0.7 |  |  | 7: MA0187.1 ... |  |
|  |  |  |  |  |  |  |  |  |  |  |  |  |  |  |  |  |  |  |  |  |  |  |  |  |  |  |  |  |  |  |  |  |  |  |  |  |  | 204 | 28 | 42.5 |  |  | 12: pTH10015 ... |  |
|  |  |  |  |  |  |  |  |  |  |  |  |  |  |  |  |  |  |  |  |  |  |  |  |  |  |  |  |  |  |  |  |  |  |  |  |  |  | 205 | 117 | 130.3 |  | 10: WBPaper00036286:Pattern B... | 6: pTH9262 ... | 58: EFL-1\_Young-adult... |
|  |  |  |  |  |  |  |  |  |  |  |  |  |  |  |  |  |  |  |  |  |  |  |  |  |  |  |  |  |  |  |  |  |  |  |  |  |  | 206 | 80 | 152.5 |  |  | 9: MA0481.1 ... | 34: LIN-35\_Fed-L1-stage-larvae... |
|  |  |  |  |  |  |  |  |  |  |  |  |  |  |  |  |  |  |  |  |  |  |  |  |  |  |  |  |  |  |  |  |  |  |  |  |  |  | 207 | 79 | 282.5 |  |  | 1: pTH0978 ... |  |
|  |  |  |  |  |  |  |  |  |  |  |  |  |  |  |  |  |  |  |  |  |  |  |  |  |  |  |  |  |  |  |  |  |  |  |  |  |  | 208 | 106 | 332.2 |  | 15: TGF-beta adult downregulated... | 52: pTH3846 ... | 45: CEH-39\_Embryos... |
|  |  |  |  |  |  |  |  |  |  |  |  |  |  |  |  |  |  |  |  |  |  |  |  |  |  |  |  |  |  |  |  |  |  |  |  |  |  | 209 | 822 | 178.1 |  | 87: TGF-beta adult downregulated... | 262: Zfp161\_2858 ... | 115: EFL-1\_Fed-L1-stage-larvae... |
|  |  |  |  |  |  |  |  |  |  |  |  |  |  |  |  |  |  |  |  |  |  |  |  |  |  |  |  |  |  |  |  |  |  |  |  |  |  | 210 | 45 | 2 |  |  |  |  |
|  |  |  |  |  |  |  |  |  |  |  |  |  |  |  |  |  |  |  |  |  |  |  |  |  |  |  |  |  |  |  |  |  |  |  |  |  |  | 211 | 54 | 89.3 |  |  | 1: V$PAX5\_01 ... | 7: DPL-1\_Young-adult... |
|  |  |  |  |  |  |  |  |  |  |  |  |  |  |  |  |  |  |  |  |  |  |  |  |  |  |  |  |  |  |  |  |  |  |  |  |  |  | 212 | 285 | 248.2 | 2: nerve ring... | 44: AFD AWB vs unsorted upregulated... | 618: pTH8982 ... | 92: C34F6.9\_Larvae-L2-stage... |
|  |  |  |  |  |  |  |  |  |  |  |  |  |  |  |  |  |  |  |  |  |  |  |  |  |  |  |  |  |  |  |  |  |  |  |  |  |  | 213 | 87 | 187.5 |  |  | 8: pTH8985 ... | 13: LSY-2\_Embryos... |
|  |  |  |  |  |  |  |  |  |  |  |  |  |  |  |  |  |  |  |  |  |  |  |  |  |  |  |  |  |  |  |  |  |  |  |  |  |  | 214 | 75 | 116.3 |  |  | 16: MA0536.1 ... | 71: EFL-1\_Fed-L1-stage-larvae... |
|  |  |  |  |  |  |  |  |  |  |  |  |  |  |  |  |  |  |  |  |  |  |  |  |  |  |  |  |  |  |  |  |  |  |  |  |  |  | 215 | 56 | 69.9 |  |  | 2: MA0095.2 ... | 1: FKH-2\_Larvae-L3-stage... |
|  |  |  |  |  |  |  |  |  |  |  |  |  |  |  |  |  |  |  |  |  |  |  |  |  |  |  |  |  |  |  |  |  |  |  |  |  |  | 216 | 62 | 135 |  |  | 11: pTH9884 ... | 11: GEI-11\_Fed-L1-stage-larvae... |
|  |  |  |  |  |  |  |  |  |  |  |  |  |  |  |  |  |  |  |  |  |  |  |  |  |  |  |  |  |  |  |  |  |  |  |  |  |  | 217 | 72 | 289.7 |  |  | 13: pTH9159 ... | 3: NHR-28\_Larvae-L4-stage... |
|  |  |  |  |  |  |  |  |  |  |  |  |  |  |  |  |  |  |  |  |  |  |  |  |  |  |  |  |  |  |  |  |  |  |  |  |  |  | 218 | 74 | 62.1 |  |  | 5: Mw140 ... |  |
|  |  |  |  |  |  |  |  |  |  |  |  |  |  |  |  |  |  |  |  |  |  |  |  |  |  |  |  |  |  |  |  |  |  |  |  |  |  | 219 | 155 | 108.7 |  |  | 5: MA0536.1 ... | 23: EFL-1\_Fed-L1-stage-larvae... |
|  |  |  |  |  |  |  |  |  |  |  |  |  |  |  |  |  |  |  |  |  |  |  |  |  |  |  |  |  |  |  |  |  |  |  |  |  |  | 220 | 224 | 272.2 | 7: reproductive system... | 20: FBF-1 Associated... | 451: ONECUT1\_2 ... | 131: CES-1\_Embryos... |
|  |  |  |  |  |  |  |  |  |  |  |  |  |  |  |  |  |  |  |  |  |  |  |  |  |  |  |  |  |  |  |  |  |  |  |  |  |  | 221 | 141 | 56.9 |  | 6: hypodermis embryo selectively enriched... | 411: klu\_SOLEXA\_5\_FBgn0013469 ... |  |
|  |  |  |  |  |  |  |  |  |  |  |  |  |  |  |  |  |  |  |  |  |  |  |  |  |  |  |  |  |  |  |  |  |  |  |  |  |  | 222 | 321 | 56.6 |  | 30: hypodermis embryo enriched... | 272: GATA3\_1 ... | 16: NHR-28\_Larvae-L4-stage... |
|  |  |  |  |  |  |  |  |  |  |  |  |  |  |  |  |  |  |  |  |  |  |  |  |  |  |  |  |  |  |  |  |  |  |  |  |  |  | 223 | 56 | 480.6 |  | 43: hypodermis embryo enriched... | 155: NR2F1\_3 ... | 13: ELT-1\_Larvae-L2-stage... |
|  |  |  |  |  |  |  |  |  |  |  |  |  |  |  |  |  |  |  |  |  |  |  |  |  |  |  |  |  |  |  |  |  |  |  |  |  |  | 224 | 33 | 7.7 |  |  | 5: Mw142 ... |  |
|  |  |  |  |  |  |  |  |  |  |  |  |  |  |  |  |  |  |  |  |  |  |  |  |  |  |  |  |  |  |  |  |  |  |  |  |  |  | 225 | 146 | 0.2 |  |  | 7: Six6\_2267 ... |  |
|  |  |  |  |  |  |  |  |  |  |  |  |  |  |  |  |  |  |  |  |  |  |  |  |  |  |  |  |  |  |  |  |  |  |  |  |  |  | 226 | 55 | 1.4 |  |  | 5: MA0499.1 ... |  |
|  |  |  |  |  |  |  |  |  |  |  |  |  |  |  |  |  |  |  |  |  |  |  |  |  |  |  |  |  |  |  |  |  |  |  |  |  |  | 227 | 28 | 23.2 |  |  | 2: FOXJ3\_1 ... |  |
|  |  |  |  |  |  |  |  |  |  |  |  |  |  |  |  |  |  |  |  |  |  |  |  |  |  |  |  |  |  |  |  |  |  |  |  |  |  | 228 | 65 | 1.2 |  |  |  | 1: NHR-77\_Fed-L1-stage-larvae... |
|  |  |  |  |  |  |  |  |  |  |  |  |  |  |  |  |  |  |  |  |  |  |  |  |  |  |  |  |  |  |  |  |  |  |  |  |  |  | 229 | 25 | 10.5 |  |  | 5: NR4A1\_f1 ... |  |
|  |  |  |  |  |  |  |  |  |  |  |  |  |  |  |  |  |  |  |  |  |  |  |  |  |  |  |  |  |  |  |  |  |  |  |  |  |  | 230 | 20 | 0.4 |  |  |  |  |
|  |  |  |  |  |  |  |  |  |  |  |  |  |  |  |  |  |  |  |  |  |  |  |  |  |  |  |  |  |  |  |  |  |  |  |  |  |  | 231 | 28 | 3.1 |  |  | 5: V$RFX1\_01 ... |  |
|  |  |  |  |  |  |  |  |  |  |  |  |  |  |  |  |  |  |  |  |  |  |  |  |  |  |  |  |  |  |  |  |  |  |  |  |  |  | 232 | 53 | 7.4 |  |  | 11: Meis3\_2 ... |  |
|  |  |  |  |  |  |  |  |  |  |  |  |  |  |  |  |  |  |  |  |  |  |  |  |  |  |  |  |  |  |  |  |  |  |  |  |  |  | 233 | 226 | 2.1 |  |  | 6: ELK3\_f1 ... |  |
|  |  |  |  |  |  |  |  |  |  |  |  |  |  |  |  |  |  |  |  |  |  |  |  |  |  |  |  |  |  |  |  |  |  |  |  |  |  | 234 | 107 | 103.5 |  |  | 35: HepG2b\_TR4\_UCD ... | 43: W03F9.2\_L4-Young-Adult-stage-larvae... |
|  |  |  |  |  |  |  |  |  |  |  |  |  |  |  |  |  |  |  |  |  |  |  |  |  |  |  |  |  |  |  |  |  |  |  |  |  |  | 235 | 217 | 469.9 | 14: intestine... | 34: CE X.nematophila regulated... | 864: CG2052\_SANGER\_2.5\_FBgn0039905 ... | 130: EFL-1\_Young-adult... |
|  |  |  |  |  |  |  |  |  |  |  |  |  |  |  |  |  |  |  |  |  |  |  |  |  |  |  |  |  |  |  |  |  |  |  |  |  |  | 236 | 472 | 277.5 |  | 37: FBF-1 Associated... | 258: ONECUT1\_1 ... | 133: EFL-1\_Young-adult... |
|  |  |  |  |  |  |  |  |  |  |  |  |  |  |  |  |  |  |  |  |  |  |  |  |  |  |  |  |  |  |  |  |  |  |  |  |  |  | 237 | 94 | 4 |  |  | 17: NR2F6\_2 ... |  |
|  |  |  |  |  |  |  |  |  |  |  |  |  |  |  |  |  |  |  |  |  |  |  |  |  |  |  |  |  |  |  |  |  |  |  |  |  |  | 238 | 127 | 22.3 |  |  | 43: STF1\_f1 ... |  |
|  |  |  |  |  |  |  |  |  |  |  |  |  |  |  |  |  |  |  |  |  |  |  |  |  |  |  |  |  |  |  |  |  |  |  |  |  |  | 239 | 190 | 210.7 |  | 20: alg-1(gk214) downregulated... | 17: MA0536.1 ... | 56: EFL-1\_Fed-L1-stage-larvae... |
|  |  |  |  |  |  |  |  |  |  |  |  |  |  |  |  |  |  |  |  |  |  |  |  |  |  |  |  |  |  |  |  |  |  |  |  |  |  | 240 | 54 | 39.9 |  |  | 3: pTH8411 ... |  |
|  |  |  |  |  |  |  |  |  |  |  |  |  |  |  |  |  |  |  |  |  |  |  |  |  |  |  |  |  |  |  |  |  |  |  |  |  |  | 241 | 98 | 124 |  | 15: TGF-beta adult downregulated... | 7: V$NKX61\_01 ... | 40: EFL-1\_Fed-L1-stage-larvae... |
|  |  |  |  |  |  |  |  |  |  |  |  |  |  |  |  |  |  |  |  |  |  |  |  |  |  |  |  |  |  |  |  |  |  |  |  |  |  | 242 | 395 | 242.6 |  | 64: alg-1(gk214) downregulated... | 547: pTH3796 ... | 119: EFL-1\_Fed-L1-stage-larvae... |
|  |  |  |  |  |  |  |  |  |  |  |  |  |  |  |  |  |  |  |  |  |  |  |  |  |  |  |  |  |  |  |  |  |  |  |  |  |  | 243 | 55 | 0.1 |  |  |  | 2: MED-1\_Embryos... |
|  |  |  |  |  |  |  |  |  |  |  |  |  |  |  |  |  |  |  |  |  |  |  |  |  |  |  |  |  |  |  |  |  |  |  |  |  |  | 244 | 40 | 136.4 |  | 11: Larva Pan Neuronal Enriched... | 1253: MA0467.1 ... | 4: CES-1\_Larvae-L3-stage... |
|  |  |  |  |  |  |  |  |  |  |  |  |  |  |  |  |  |  |  |  |  |  |  |  |  |  |  |  |  |  |  |  |  |  |  |  |  |  | 245 | 376 | 175 | 57: lateral pharyngeal ganglion right neuron... | 33: Larval Pan Neuronal... | 1801: pTH10722 ... | 10: ALR-1\_Larvae-L2-stage... |
|  |  |  |  |  |  |  |  |  |  |  |  |  |  |  |  |  |  |  |  |  |  |  |  |  |  |  |  |  |  |  |  |  |  |  |  |  |  | 246 | 57 | 194.7 |  | 1: up in zfp-1... | 68: pTH6556 ... | 18: ZTF-4\_Larvae-L2-stage... |
|  |  |  |  |  |  |  |  |  |  |  |  |  |  |  |  |  |  |  |  |  |  |  |  |  |  |  |  |  |  |  |  |  |  |  |  |  |  | 247 | 34 | 0.8 |  |  | 5: exd\_SOLEXA\_2\_FBgn0000611 ... |  |
|  |  |  |  |  |  |  |  |  |  |  |  |  |  |  |  |  |  |  |  |  |  |  |  |  |  |  |  |  |  |  |  |  |  |  |  |  |  | 248 | 224 | 9.5 |  | 6: mir-35 downregulated... | 105: Gata6\_3769 ... | 8: NHR-28\_Larvae-L4-stage... |
|  |  |  |  |  |  |  |  |  |  |  |  |  |  |  |  |  |  |  |  |  |  |  |  |  |  |  |  |  |  |  |  |  |  |  |  |  |  | 249 | 56 | 20.8 |  |  | 4: pTH9322 ... |  |
|  |  |  |  |  |  |  |  |  |  |  |  |  |  |  |  |  |  |  |  |  |  |  |  |  |  |  |  |  |  |  |  |  |  |  |  |  |  | 250 | 131 | 16.5 | 13: PQR... | 7: Larval Pan Neuronal... | 379: pTH9226 ... |  |
|  |  |  |  |  |  |  |  |  |  |  |  |  |  |  |  |  |  |  |  |  |  |  |  |  |  |  |  |  |  |  |  |  |  |  |  |  |  | 251 | 320 | 27.7 |  | 169: intestine embryo enriched... | 166: srp\_SANGER\_5\_FBgn0003507 ... | 20: FOS-1\_Larvae-L2-stage... |
|  |  |  |  |  |  |  |  |  |  |  |  |  |  |  |  |  |  |  |  |  |  |  |  |  |  |  |  |  |  |  |  |  |  |  |  |  |  | 252 | 160 | 10.7 |  | 46: intestine larva enriched... | 101: pTH10707 ... | 11: FOS-1\_Larvae-L2-stage... |
|  |  |  |  |  |  |  |  |  |  |  |  |  |  |  |  |  |  |  |  |  |  |  |  |  |  |  |  |  |  |  |  |  |  |  |  |  |  | 253 | 37 | 76.8 |  |  | 7: RORA\_2 ... |  |
|  |  |  |  |  |  |  |  |  |  |  |  |  |  |  |  |  |  |  |  |  |  |  |  |  |  |  |  |  |  |  |  |  |  |  |  |  |  | 254 | 172 | 0.2 |  |  | 9: Meis2\_1 ... |  |
|  |  |  |  |  |  |  |  |  |  |  |  |  |  |  |  |  |  |  |  |  |  |  |  |  |  |  |  |  |  |  |  |  |  |  |  |  |  | 255 | 40 | 0.4 |  |  | 1: CENPB\_1 ... |  |
|  |  |  |  |  |  |  |  |  |  |  |  |  |  |  |  |  |  |  |  |  |  |  |  |  |  |  |  |  |  |  |  |  |  |  |  |  |  | 256 | 92 | 8.3 |  |  | 119: MA0262.1 ... | 3: NHR-77\_Larvae-L4-stage... |
|  |  |  |  |  |  |  |  |  |  |  |  |  |  |  |  |  |  |  |  |  |  |  |  |  |  |  |  |  |  |  |  |  |  |  |  |  |  | 257 | 114 | 126.2 |  | 5: 0hr muscle enriched... | 660: FOXJ2\_f1 ... | 11: PHA-4\_Larvae-L2-stage... |
|  |  |  |  |  |  |  |  |  |  |  |  |  |  |  |  |  |  |  |  |  |  |  |  |  |  |  |  |  |  |  |  |  |  |  |  |  |  | 258 | 73 | 93.5 |  | 4: WBPaper00036286:Pattern C... | 163: MA0481.1 ... | 1: NHR-28\_Larvae-L4-stage... |
|  |  |  |  |  |  |  |  |  |  |  |  |  |  |  |  |  |  |  |  |  |  |  |  |  |  |  |  |  |  |  |  |  |  |  |  |  |  | 259 | 68 | 105.3 | 5: anterior ganglion... | 3: GABAergic-motor-neurons embryo selectively enriched... | 607: Nkx2-9\_3082 ... |  |
|  |  |  |  |  |  |  |  |  |  |  |  |  |  |  |  |  |  |  |  |  |  |  |  |  |  |  |  |  |  |  |  |  |  |  |  |  |  | 260 | 55 | 83.6 |  | 4: all-neurons larva selectively enriched... | 1613: MA0165.1 ... | 1: ALR-1\_Larvae-L2-stage... |
|  |  |  |  |  |  |  |  |  |  |  |  |  |  |  |  |  |  |  |  |  |  |  |  |  |  |  |  |  |  |  |  |  |  |  |  |  |  | 261 | 203 | 1.8 |  |  |  | 1: NHR-12\_Larvae-L3-stage... |
|  |  |  |  |  |  |  |  |  |  |  |  |  |  |  |  |  |  |  |  |  |  |  |  |  |  |  |  |  |  |  |  |  |  |  |  |  |  | 262 | 25 | 84.7 |  |  | 7: pTH9928 ... |  |
|  |  |  |  |  |  |  |  |  |  |  |  |  |  |  |  |  |  |  |  |  |  |  |  |  |  |  |  |  |  |  |  |  |  |  |  |  |  | 263 | 13 | 0.5 |  |  | 2: SMAD3\_1 ... |  |
|  |  |  |  |  |  |  |  |  |  |  |  |  |  |  |  |  |  |  |  |  |  |  |  |  |  |  |  |  |  |  |  |  |  |  |  |  |  | 264 | 128 | 3 |  |  |  |  |
|  |  |  |  |  |  |  |  |  |  |  |  |  |  |  |  |  |  |  |  |  |  |  |  |  |  |  |  |  |  |  |  |  |  |  |  |  |  | 265 | 71 | 0.6 |  |  | 5: pTH10650 ... |  |
|  |  |  |  |  |  |  |  |  |  |  |  |  |  |  |  |  |  |  |  |  |  |  |  |  |  |  |  |  |  |  |  |  |  |  |  |  |  | 266 | 36 | 0.7 |  |  | 9: crp\_SANGER\_10\_FBgn0001994 ... |  |
|  |  |  |  |  |  |  |  |  |  |  |  |  |  |  |  |  |  |  |  |  |  |  |  |  |  |  |  |  |  |  |  |  |  |  |  |  |  | 267 | 20 | 2.8 |  |  | 1: TBX20\_1 ... |  |
|  |  |  |  |  |  |  |  |  |  |  |  |  |  |  |  |  |  |  |  |  |  |  |  |  |  |  |  |  |  |  |  |  |  |  |  |  |  | 268 | 74 | 57.4 |  | 9: WBPaper00036286:Pattern G... | 897: EN2\_1 ... |  |
|  |  |  |  |  |  |  |  |  |  |  |  |  |  |  |  |  |  |  |  |  |  |  |  |  |  |  |  |  |  |  |  |  |  |  |  |  |  | 269 | 65 | 113.7 |  | 3: 0hr muscle depleted... | 925: MA0546.1 ... |  |
|  |  |  |  |  |  |  |  |  |  |  |  |  |  |  |  |  |  |  |  |  |  |  |  |  |  |  |  |  |  |  |  |  |  |  |  |  |  | 270 | 61 | 32.9 |  | 2: all-neurons larva selectively enriched... | 88: RFX5\_2 ... |  |
|  |  |  |  |  |  |  |  |  |  |  |  |  |  |  |  |  |  |  |  |  |  |  |  |  |  |  |  |  |  |  |  |  |  |  |  |  |  | 271 | 54 | 234.7 |  |  | 1150: Dfd\_Cell\_FBgn0000439 ... | 20: HAM-1\_Fed-L1-stage-larvae... |
|  |  |  |  |  |  |  |  |  |  |  |  |  |  |  |  |  |  |  |  |  |  |  |  |  |  |  |  |  |  |  |  |  |  |  |  |  |  | 272 | 26 | 32.8 |  |  | 28: NHLH1\_2 ... |  |
|  |  |  |  |  |  |  |  |  |  |  |  |  |  |  |  |  |  |  |  |  |  |  |  |  |  |  |  |  |  |  |  |  |  |  |  |  |  | 273 | 54 | 102.8 |  |  | 726: Otp\_3496 ... |  |
|  |  |  |  |  |  |  |  |  |  |  |  |  |  |  |  |  |  |  |  |  |  |  |  |  |  |  |  |  |  |  |  |  |  |  |  |  |  | 274 | 55 | 0.8 |  |  | 4: gl\_SANGER\_5\_FBgn0004618 ... |  |
|  |  |  |  |  |  |  |  |  |  |  |  |  |  |  |  |  |  |  |  |  |  |  |  |  |  |  |  |  |  |  |  |  |  |  |  |  |  | 275 | 34 | 0.8 |  |  |  |  |
|  |  |  |  |  |  |  |  |  |  |  |  |  |  |  |  |  |  |  |  |  |  |  |  |  |  |  |  |  |  |  |  |  |  |  |  |  |  | 276 | 52 | 335.5 |  |  | 35: OTX1\_f1 ... | 1: NHR-2\_Embryos... |
|  |  |  |  |  |  |  |  |  |  |  |  |  |  |  |  |  |  |  |  |  |  |  |  |  |  |  |  |  |  |  |  |  |  |  |  |  |  | 277 | 27 | 9.5 |  |  | 5: MA0017.1 ... |  |
|  |  |  |  |  |  |  |  |  |  |  |  |  |  |  |  |  |  |  |  |  |  |  |  |  |  |  |  |  |  |  |  |  |  |  |  |  |  | 278 | 52 | 0.7 |  |  | 8: Pph13\_Cell\_FBgn0023489 ... |  |
|  |  |  |  |  |  |  |  |  |  |  |  |  |  |  |  |  |  |  |  |  |  |  |  |  |  |  |  |  |  |  |  |  |  |  |  |  |  | 279 | 27 | 0.7 |  |  | 3: MEF2D\_1 ... |  |
|  |  |  |  |  |  |  |  |  |  |  |  |  |  |  |  |  |  |  |  |  |  |  |  |  |  |  |  |  |  |  |  |  |  |  |  |  |  | 280 | 149 | 394.5 | 64: somatic nervous system... | 30: enriched in PVD OLL... | 1527: MA0543.1 ... | 117: CES-1\_Embryos... |
|  |  |  |  |  |  |  |  |  |  |  |  |  |  |  |  |  |  |  |  |  |  |  |  |  |  |  |  |  |  |  |  |  |  |  |  |  |  | 281 | 64 | 16.3 |  |  | 3: pTH10040 ... | 2: SAX-3\_Fed-L1-stage-larvae... |
|  |  |  |  |  |  |  |  |  |  |  |  |  |  |  |  |  |  |  |  |  |  |  |  |  |  |  |  |  |  |  |  |  |  |  |  |  |  | 282 | 25 | 0.4 |  |  | 2: pTH9247 ... |  |
|  |  |  |  |  |  |  |  |  |  |  |  |  |  |  |  |  |  |  |  |  |  |  |  |  |  |  |  |  |  |  |  |  |  |  |  |  |  | 283 | 48 | 22.2 |  | 3: excretory-cell larva enriched... | 37: pTH9052 ... |  |
|  |  |  |  |  |  |  |  |  |  |  |  |  |  |  |  |  |  |  |  |  |  |  |  |  |  |  |  |  |  |  |  |  |  |  |  |  |  | 284 | 371 | 244.7 | 54: somatic nervous system... | 60: Larval Pan Neuronal... | 1815: MA0543.1 ... | 69: CES-1\_Embryos... |
|  |  |  |  |  |  |  |  |  |  |  |  |  |  |  |  |  |  |  |  |  |  |  |  |  |  |  |  |  |  |  |  |  |  |  |  |  |  | 285 | 48 | 24.1 |  | 1: A-class-motor-neurons larva selectively enriched... | 242: I$KR\_01 ... |  |
|  |  |  |  |  |  |  |  |  |  |  |  |  |  |  |  |  |  |  |  |  |  |  |  |  |  |  |  |  |  |  |  |  |  |  |  |  |  | 286 | 170 | 116.7 | 185: phasmid neuron... | 22: BAG-neuron embryo enriched... | 82: RFX3\_1 ... |  |
|  |  |  |  |  |  |  |  |  |  |  |  |  |  |  |  |  |  |  |  |  |  |  |  |  |  |  |  |  |  |  |  |  |  |  |  |  |  | 287 | 81 | 137.1 |  | 8: Larval Pan Neuronal... | 1000: nau\_da\_SANGER\_5\_FBgn0002922 ... | 1: C34F6.9\_Larvae-L2-stage... |
|  |  |  |  |  |  |  |  |  |  |  |  |  |  |  |  |  |  |  |  |  |  |  |  |  |  |  |  |  |  |  |  |  |  |  |  |  |  | 288 | 126 | 28.5 |  |  | 31: Hoxa2\_1 ... |  |
|  |  |  |  |  |  |  |  |  |  |  |  |  |  |  |  |  |  |  |  |  |  |  |  |  |  |  |  |  |  |  |  |  |  |  |  |  |  | 289 | 74 | 44.2 |  |  | 23: PAX6\_1 ... | 1: NHR-12\_Larvae-L3-stage... |
|  |  |  |  |  |  |  |  |  |  |  |  |  |  |  |  |  |  |  |  |  |  |  |  |  |  |  |  |  |  |  |  |  |  |  |  |  |  | 290 | 70 | 39.6 |  |  | 23: TFAP4\_2 ... | 2: UNC-62\_Day-Four-Young-Adult... |
|  |  |  |  |  |  |  |  |  |  |  |  |  |  |  |  |  |  |  |  |  |  |  |  |  |  |  |  |  |  |  |  |  |  |  |  |  |  | 291 | 72 | 31.7 |  |  | 97: ALX4\_1 ... |  |
|  |  |  |  |  |  |  |  |  |  |  |  |  |  |  |  |  |  |  |  |  |  |  |  |  |  |  |  |  |  |  |  |  |  |  |  |  |  | 292 | 139 | 2.7 |  |  | 7: MA0118.1 ... |  |
|  |  |  |  |  |  |  |  |  |  |  |  |  |  |  |  |  |  |  |  |  |  |  |  |  |  |  |  |  |  |  |  |  |  |  |  |  |  | 293 | 58 | 0.8 |  |  | 9: Hmx1\_3423 ... |  |
|  |  |  |  |  |  |  |  |  |  |  |  |  |  |  |  |  |  |  |  |  |  |  |  |  |  |  |  |  |  |  |  |  |  |  |  |  |  | 294 | 25 | 71.9 |  |  | 9: pTH10038 ... |  |
|  |  |  |  |  |  |  |  |  |  |  |  |  |  |  |  |  |  |  |  |  |  |  |  |  |  |  |  |  |  |  |  |  |  |  |  |  |  | 295 | 58 | 479.5 |  | 8: Larval Pan Neuronal... | 1241: ALX3\_3 ... | 17: CES-1\_Fed-L1-stage-larvae... |
|  |  |  |  |  |  |  |  |  |  |  |  |  |  |  |  |  |  |  |  |  |  |  |  |  |  |  |  |  |  |  |  |  |  |  |  |  |  | 296 | 38 | 0.9 |  |  | 6: pTH9225 ... | 3: FOS-1\_Larvae-L2-stage... |
|  |  |  |  |  |  |  |  |  |  |  |  |  |  |  |  |  |  |  |  |  |  |  |  |  |  |  |  |  |  |  |  |  |  |  |  |  |  | 297 | 58 | 31.8 |  | 1: [cgc6390]:male germline-enriched... | 2: MA0067.1 ... |  |
|  |  |  |  |  |  |  |  |  |  |  |  |  |  |  |  |  |  |  |  |  |  |  |  |  |  |  |  |  |  |  |  |  |  |  |  |  |  | 298 | 52 | 88.7 |  | 3: [cgc5767]:cluster 52... | 1: pTH10624 ... | 4: AHA-1\_Fed-L1-stage-larvae... |
|  |  |  |  |  |  |  |  |  |  |  |  |  |  |  |  |  |  |  |  |  |  |  |  |  |  |  |  |  |  |  |  |  |  |  |  |  |  | 299 | 65 | 35.7 | 6: sensory neuron... | 10: all-neurons larva enriched... | 241: Sox15\_3457 ... |  |
|  |  |  |  |  |  |  |  |  |  |  |  |  |  |  |  |  |  |  |  |  |  |  |  |  |  |  |  |  |  |  |  |  |  |  |  |  |  | 300 | 172 | 11.6 |  | 3: coelomocytes embryo selectively enriched... | 23: MA0040.1 ... |  |
